# Antiproliferative effects of sapacitabine (CYC682), a novel 2′-deoxycytidine-derivative, in human cancer cells

**DOI:** 10.1038/sj.bjc.6603896

**Published:** 2007-07-17

**Authors:** M Serova, C M Galmarini, A Ghoul, K Benhadji, S R Green, J Chiao, S Faivre, E Cvitkovic, C Le Tourneau, F Calvo, E Raymond

**Affiliations:** 1 RayLab – Department of Medical Oncology, Hôpital Beaujon, 100 boulevard Général Leclerc, Clichy 92110, France; 2 Laboratoire de Pharmacologie Expérimentale et Clinique U716 IGM – Hôpital Saint-Louis, 27 rue Juliette Dodu, Paris 75010, France; 3 ENS-CNRS UMR 5239, U.F.R. de Médecine Lyon-Sud, 165 chemin du Grand Revoyet, BP12, Oullins Cedex 69921, France; 4 Department of Clinical Pharmacology, Centre René Huguenin, 35 Rue Dailly, Saint-Cloud 92210, France; 5 Cyclacel Ltd, James Lindsay Place, Dundee, DD1 5JJ, UK; 6 AAI Oncology, 18-20 rue Pasteur, Le Kremlin Bicetre, 94278, France

**Keywords:** CS-682, nucleoside analogue, combination index, synergy, antimetabolites

## Abstract

This study assessed the antiproliferative activity of sapacitabine (CYC682, CS-682) in a panel of 10 human cancer cell lines with varying degrees of resistance or sensitivity to the commonly used nucleoside analogues ara-C and gemcitabine. Growth inhibition studies using sapacitabine and CNDAC were performed in the panel of cell lines and compared with both nucleoside analogues and other anticancer compounds including oxaliplatin, doxorubicin, docetaxel and seliciclib. Sapacitabine displayed antiproliferative activity across a range of concentrations in a variety of cell lines, including those shown to be resistant to several anticancer drugs. Sapacitabine is biotransformed by plasma, gut and liver amidases into CNDAC and causes cell cycle arrest predominantly in the G_2_/M phase. No clear correlation was observed between sensitivity to sapacitabine and the expression of critical factors involved in resistance to nucleoside analogues such as deoxycytidine kinase (dCK), human equilibrative nucleoside transporter 1, cytosolic 5′-nucleotidase and DNA polymerase-α. However, sapacitabine showed cytotoxic activity against dCK-deficient L1210 cells indicating that in some cells, a dCK-independent mechanism of action may be involved. In addition, sapacitabine showed a synergistic effect when combined with gemcitabine and sequence-specific synergy with doxorubicin and oxaliplatin. Sapacitabine is therefore a good candidate for further evaluation in combination with currently used anticancer agents in tumour types with unmet needs.

Sapacitabine (Figure 1A; CYC682, Cyclacel Ltd, Dundee, UK; CS-682, Sankyo, Tokyo, Japan) is a novel 2′-deoxycytidine analogue (1-(2-C-cyano-2-deoxy-*β*-D-arabino-pentafuranosyl)N4-palmitoylcytosine), which was synthesised by incorporating an additional N4-palmitoyl group to protect the amino group (Kaneko *et al*, 1997). The rational of adding a N4-palmitoyl moiety to CNDAC (1-(2-C-cyano-2-deoxy-*β*-D-arabino-pentafuranosyl) cytosine) resulting in sapacitabine was expected to protect the amino group of CNDAC, resulting in a better diffusion into gastro-intestinal cells thereby allowing the oral administration of the drug. Sapacitabine is primarily metabolised by plasma, gut and liver amidases into the active metabolite CNDAC, which is subsequently transported inside cells and then phosphorylated by deoxycytidine kinase (dCK) into CNDAC triphosphate before being incorporated into cellular DNA (Figure 1B). Extension during DNA replication leads to single-strand breaks directly caused by *β*-elimination (Liu *et al*, 2005). DNA strand breaks that arise from further processing initiate signals that activate the G_2_ checkpoint pathway, ultimately resulting in cellular apoptosis. After incubation with cytostatic concentrations of CNDAC, cell cycle arrest in G_2_ occurs following a delayed S phase (Liu *et al*, 2005). This differs from other deoxycytidine analogues such as ara-C or gemcitabine for which the predominant biological alterations consist of cell cycle arrest in S phase (Azuma *et al*, 2001). Cytotoxic effects of CNDAC have also been associated with intracellular accumulation of CNDAC triphosphate and chain termination (Galmarini, 2006).

Although they have a similar mechanism of action, preliminary *in vitro* and *in vivo* investigations have shown that CNDAC and sapacitabine may display overlapping cytotoxic effects with some cancer cells being more sensitive to sapacitabine than CNDAC. Furthermore, data have suggested that the sapacitabine cytotoxicity profile may differ from that of other nucleoside analogues (Kaneko *et al*, 1997; Faivre *et al*, 1999; Hanaoka *et al*, 1999; Zhang *et al*, 1999; Wu *et al*, 2003; Matsuda and Sasaki, 2004). Therefore, further investigations are required to ascertain the exact mechanism of action of sapacitabine, to evaluate the toxicity of combinations with other compounds and to screen the range of its activity in various tumour systems that may eventually lead to clinical application.

In this study, we tested the antiproliferative activity and cell cycle effects of sapacitabine against a panel of colon, breast, lung and ovarian cancer cell lines considered as being representative of tumours warranting the development of this novel anticancer agent. The antiproliferative effects of sapacitabine were observed in a variety of cell lines resistant to other anticancer drugs. Levels of mRNA expression of critical factors including various transporters and enzymes involved in resistance to nucleoside analogues, such as dCK, human equilibrative nucleoside transporter 1 (hENT1), cytosolic 5′-nucleotidase (cN-II) and DNA polymerase-*α* (POL), were determined in all cell lines and correlated with sapacitabine cytotoxicity. Sapacitabine also showed cytotoxic activity against dCK-deficient L1210 cells.

Considering that this compound may be amenable to clinical trials in combination with other anticancer agents, combinations of sapacitabine with several anticancer agents commonly used in practice, such as gemcitabine, oxaliplatin, doxorubicin, docetaxel and seliciclib, a novel CDK inhibitor (McClue *et al*, 2002; Meijer and Raymond, 2003), were also explored.

## MATERIALS AND METHODS

### Cell lines

A panel of colon (HT29, HCT116, COLO205, HCC2998), breast (MCF7, MDA-MB-435), lung (HOP62, HOP92), ovarian (OVCAR3, IGROV1) cancer cell lines was purchased from the ATCC (Rockville, MD, USA). Cells were grown as monolayers in RPMI medium supplemented with 10% fetal calf serum (Invitrogen, Cergy-Pontoise, France), 2 mM glutamine, 100 units ml^−1^ penicillin and 100 *μ*M ml^−1^ streptomycin.

### Cell cytotoxicity assays

All the data generated were the result of three separate experiments performed in duplicate. Cell viability was determined using the MTT assay, which was carried out as described previously (Hansen *et al*, 1989). Briefly, cells were seeded in 96-well plates at a density of 2 × 10^3^ cells per well. Cells were incubated for 120 h and then 0.4 mg ml^−1^ of MTT dye (3-[4, 5-dimethylthiazol-2-yl]-2, 5-diphenyltetrazolium bromide; Sigma, Saint-Quentin Fallavier, France) was added for 4 h at 37°C. The monolayer was suspended in 0.1 ml of DMSO and the absorbance at 560 nm was measured using a microplate reader (Dynatech, Ann Arbor, MI, USA). Positive and negative controls included wells with untreated cells or medium containing MTT with no cells, respectively. The conversion of yellow water-soluble tetrazolium MTT into purple insoluble formazan is catalysed by mitochondrial dehydrogenases and is used to estimate the number of viable cells. The control value corresponding to untreated cells was taken as 100% and the viability of treated samples was expressed as a percentage of the control. IC_50_ values were determined as concentrations that reduced cell viability by 50%.

For single agent studies, cells were seeded and allowed to settle for 24 h before treatment with increasing concentrations of sapacitabine, CNDAC, seliciclib (CYC202, R-roscovitine; Meijer and Raymond, 2003), a novel cyclin dependent kinase inhibitor, doxorubicin, docetaxel, cisplatin, oxaliplatin, ara-C, 5-FU or gemcitabine for 48 h. After incubation, the cells were allowed to recover in compound-free medium for 72 h, before determination of growth inhibition using the MTT assay.

For sequential or simultaneous studies, three schedules were implemented comprising sequential exposure to sapacitabine (48 h) followed by the second agent (24 h); sequential exposure with one anticancer agent (24 h) followed by treatment with sapacitabine (for 48 h); and simultaneous exposure to both agents (48 h). In sequential exposure schedules, cells were seeded and allowed to grow in the presence of various concentrations of sapacitabine, doxorubicin, docetaxel, seliciclib, oxaliplatin or gemcitabine for 24 or 48 h. The supernatant was then removed and the second compound was added. After an additional exposure period, the second compound was removed and cells were allowed to recover in drug-free medium for 72 h. Growth inhibition was then determined by the MTT assay. For simultaneous exposure, cells were seeded and treated after 24 h with increasing concentrations of sapacitabine alone or with seliciclib, doxorubicin, docetaxel, oxaliplatin or gemcitabine in various concentrations corresponding to the IC_20_, IC_40_, IC_60_ and IC_80_ values. After approximately four doubling times (120 h), the growth inhibitory effects were measured using the MTT assay.

### Statistical analysis and determination of synergistic activity

Drug combination effects were determined using the Chou and Talalay method as described elsewhere (Chou and Talalay, 1984) based on the median effect principle. Combination index (CI) values of <1 indicate synergy, a value of 1 indicates additive effects and a value of >1 indicates antagonism. Variability between experiments led us to consider that CI values ranging from 0.8 to 1.2 mainly represent additive effects. Thereby calculation of a CI below 0.8 is an indication of synergy, above 1.2 antagonism and between 0.8 and 1.2 an indication of additive effects.

Data were analysed using the concentration effect analysis software (Biosoft, Cambridge, UK). For statistical analysis and graphs, the Instat and Prism software (GraphPad, San Diego, CA, USA) were used. Experiments were performed three times, in duplicate. Means and standard deviations were compared using Student's *t*-test (two-sided *P*-value).

### Cell cycle analysis and apoptosis

The cell cycle stage and percentage of apoptotic cells were assessed by flow cytometry. In brief, cells were seeded in 25 cm^3^ flasks and were untreated or treated with various concentrations of sapacitabine. At the indicated time points, adherent and non-adherent cells were collected, washed with PBS, fixed in 70% ethanol and stored at 4°C until use. Cells were re-hydrated in PBS, incubated for 20 min at room temperature (25°C) with 250 *μ*g ml^−1^ RNAse A with Triton X-100 and 20 min at 4°C with 50 *μ*g ml^−1^ propidium iodide in the dark. The cell cycle distribution and percentage of apoptotic cells were determined with FACScan flow cytometer and analysed by FACS Calibur (Becton Dickinson, Le-Ponte-de-Claix, France).

### RNA extraction, RT–PCR and quantitative PCR

Expression levels of the various metabolic factors involved in ara-C resistance were assessed by quantitative real-time RT–PCR performed in a LightCycler detection system (Roche, Mannheim, Germany). Briefly, cDNA (5 *μ*l) was mixed with primers (300 nM each), LightCycler-FastStart DNA Master SYBR Green I (hENT1 transporter and POL) or LightCycler-FastStart DNA master hybridisation probes (18S, dCK and cN-II), and probes (130 nM; if necessary) in a total volume of 20 *μ*l. These reactions were prepared in duplicate in three separate experiments. The primer sequences used were:

hENT1: (FOR: gctgggtctgaccgttgtat; REV: ctgtacagggtgcatgatgg);
dCK: (FOR: aaacctgaacgatggtctttttacc; REV: ctttgagcttgccattcagaga);
cN-II: (FOR: acctgctgtattaccctttcagcta; REV: gctccaccgttgattcatga);
POL: (FOR: agcttgacctgattgctgtc; REV: atgacgggacaaagacaagg).

The relative amount of each target gene and a reference gene (18S) were determined for all samples and the calibrator cell line, HL-60. Ratio results obtained with RelQuant software were considered as final relative PCR arbitrary units. Results were then expressed as PCR arbitrary units in all cell lines related to HL-60 cells PCR arbitrary unit expression.

## RESULTS

### Single agent studies

The antiproliferative effect of sapacitabine was examined in a panel of 10 cancer cell lines as displayed in Table 1. Time course experiments showed that optimal antiproliferative effects were achieved when cells were exposed to sapacitabine for 48 h. Concentrations of sapacitabine required to achieve an IC_50_ ranged from 3±0.6 *μ*M for the colon cancer cell line HCT116 to 67±14 *μ*M for the breast cancer cell line MDA-MB-435.

**Figure 2:**
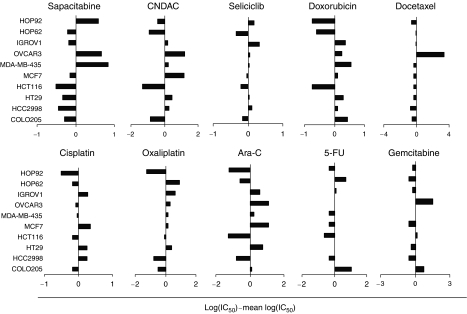
Comparative analysis of cytotoxicity of anticancer agents in a panel of human cancer cell lines. The influence of sapacitabine, CNDAC, seliciclib, doxorubicin, docetaxel, cisplatin, oxaliplatin, ara-C, 5-FU and gemcitabine on the viability of 10 different tumour cell lines was determined using the MTT assay after continuous drug exposure for four doubling times. The indicated values are calculated as follows: log (IC_50_ individual cell line)–mean (logIC_50_). Negative values indicate that the cell line is more sensitive than the average, where as positive values indicate that the cell line is more resistant than the average. The horizontal axis represents a log scale.

The antiproliferative effects of sapacitabine were compared to those of CNDAC and several commonly used anticancer agents such as gemcitabine, doxorubicin, docetaxel, oxaliplatin and seliciclib. Results are shown in Figure 2. Sapacitabine displayed cytotoxic effects against all cancer cell lines irrespective of the origin of the tumour and usually at lower concentrations than required for CNDAC. The cytotoxicity profiles of the other nucleoside analogues tested including CNDAC and ara-C appeared to differ from that of sapacitabine suggesting differences in the metabolism, mechanism of action and/or resistance between sapacitabine and other cytidine analogues.

### Effect of sapacitabine and CNDAC on cell cycle changes

**Figure 3:**
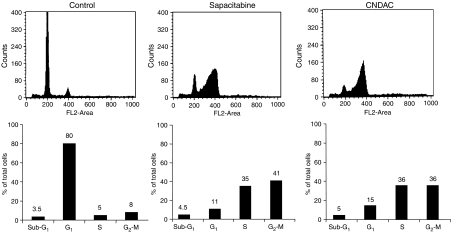
Effects of CNDAC and sapacitabine on cell cycle in HCT116 cells detected by flow cytometry. HCT116 cells were harvested after 48 h of incubation with 6 *μ*M sapacitabine or CNDAC and analysed for DNA content by FACS analysis. The lower panel represents the distribution of cells in different phases of cell cycle.

The HCT116 cell line was selected, as the most sensitive model to both sapacitabine and CNDAC, for further investigation in this study. Cells were incubated with a concentration of 6 *μ*M (twice the IC_50_) of sapacitabine or CNDAC for 48 h. Cell cycle analysis showed that 35% sapacitabine-treated cells arrested in late-S phase and 41% in G_2_/M phase. A similar pattern was observed in CNDAC-treated cells with 36 and 36% of cells arrested in late-S and G_2_/M phases, respectively (Figure 3). Thus, both agents caused the accumulation of HCT116 cells in G2/M phases as reported previously (Azuma *et al*, 2001).

### Analysis of genes involved in nucleoside analogue metabolism by quantitative real-time PCR

**Figure 4:**
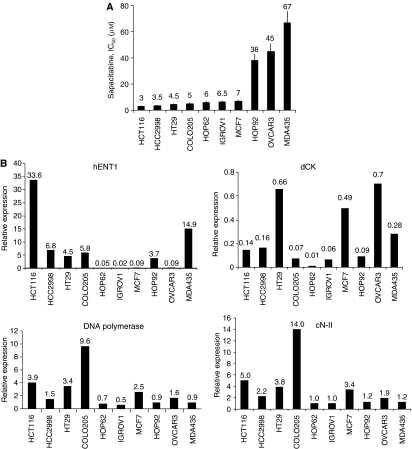
(**A**) Cytotoxicity of sapacitabine in a panel of 10 cancer cell lines. (**B**) Profiling mRNA levels of metabolic factors in 10 cell lines. Analysis of main nucleoside metabolism factors (hENT1, dCK, DNA polymerase and cN-II) was performed by quantitative real-time PCR. RNA was extracted from cells to determine transcript copy numbers relative to the reference gene 18S. Results were expressed as PCR arbitrary units in all cell lines related to HL-60 cells PCR arbitrary unit expression.

The transcription profiles of genes known to be involved in the resistance to nucleoside analogues were analysed in the panel of cancer cell lines. The mRNA levels of hENT1, dCK, cN-II and POL genes were analysed using real-time PCR (Figure 4) and calibrated against HL-60. Colorectal cell lines showed high expression of the transporter hENT1, low-medium expression of the enzyme dCK and a variable expression of the cN-II 5′-nucleotidase and POL enzymes. A similar expression profile was observed in breast cancer cell lines and the HOP92 lung cancer cell line. In contrast, the HOP62 lung cancer cell line and ovarian cancer cell lines (OVCAR3 and IGROV1) showed low expression of the transporter hENT1 and medium expression of cN-II and POL enzymes.

**Figure 5:**
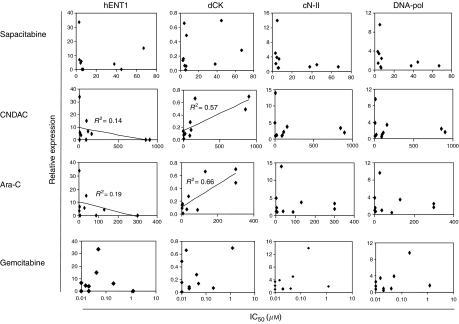
Correlation between mRNA levels and sensitivity to deoxycytidine analogues. Expression levels of hENT1, dCK, cN-II and DNA-pol mRNAs were correlated with cytotoxicity of sapacitabine, CNDAC, ara C and gemcitabine, as measured by IC_50_ values, in 10 tumour cell lines. All values from the reverse transcription-PCR are standardised with respect to HL60. The *R*^2^ value corresponds with the correlation coefficient calculated by linear regression analysis. *R*^2^<0.1 was not displayed.

Attempts were made to correlate the antiproliferative effects of sapacitabine, CNDAC, ara-C and gemcitabine with the level of mRNA expression of genes encoding the main drug metabolism proteins. As shown in Figure 5, cells with lower expression of hENT1 and cells with lower expression of dCK appear to be more resistant to both CNDAC and ara-C. In contrast, antiproliferative effects of sapacitabine and gemcitabine were not consistently associated with mRNA levels of hENT1 and dCK. Furthermore, no consistent pattern of expression of cN-II and POL genes correlated with their antiproliferative effects.

### Cytotoxicity of sapacitabine in dCK^+^/dCK^−^ leukaemia cancer cell lines

The role of dCK in resistance to ara-C has been established with a correlation observed between dCK deficiency and ara-C resistance (Galmarini *et al*, 2002). As dCK is responsible for intracellular phosphorylation of nucleosides to the active form, we evaluated the cytotoxicity of sapacitabine against the murine leukaemia cell lines L1210 and L1210dCK^−^, the latter was selected on the basis of resistance to continued passage in the presence of ara-C. L1210 cells with dCK activity were sensitive to sapacitabine, CNDAC and ara-C (IC_50_ 20±6, 1.7±0.3 and 0.08±0.02 *μ*M, respectively) (Table 1). We initially confirmed that L1210dCK^−^ cells were indeed resistant to ara-C. L1210dCK^−^ cancer cells were approximately 600- and 2500-fold more resistant to CNDAC and ara-C, respectively, than L1210 cells (IC_50_ 1000±150 and >100 *μ*M, respectively). However, L1210dCK^−^ cells remained sensitive to the parental compound sapacitabine (IC_50_ 29±4 *μ*M). Together with mRNA profiling, this suggests that sapacitabine alone has antitumour effects based on an intracellular metabolic pathway that may be independent of dCK, which in some cell lines may differ from that of ara-C and its primary metabolite CNDAC. This may be due to the action of the palmitoyl chain of sapacitabine, which is not present in CNDAC.

### Combination agent studies

Three schedules were used to study the effect of sequential and simultaneous exposure of combinations of sapacitabine with other anticancer agents. In the first schedule, sapacitabine was added to two cell lines COLO205 and HCT116 for 48 h followed by the addition of oxaliplatin, doxorubicin, docetaxel, gemcitabine or seliciclib for 24 h. The second schedule consisted of inverting the order of exposure of the cells to the different agents, and the third schedule was the simultaneous addition of sapacitabine and a second agent for a period of 48 h. Following incubation, the effect was determined using CIs that represent an affected fraction for the concentration of drugs corresponding to the IC_50_ as described previously (Chou and Talalay, 1984) (Table 2).

In the docetaxel/sapacitabine combinations, synergistic effects (CI<1) were observed when docetaxel was given before sapacitabine in both cell lines. Antagonistic effects (CI>1) were observed for COLO205 when docetaxel was given after and concomitantly to sapacitabine. In the HCT116 cell line, an additive effect was observed for sapacitabine → docetaxel schedule and an additive/synergistic effect in schedule sapacitabine+docetaxel (Table 2). Docetaxel given before sapacitabine appears as more effective in both cell lines than other schedules.

In the gemcitabine/sapacitabine combinations, synergism was observed in both cell lines with schedule gemcitabine before sapacitabine, in contrast with the antagonistic effect observed when the compounds were administered concomitantly or when sapacitabine was administered before gemcitabine. The synergism observed between sapacitabine and gemcitabine suggests that, although closely related, those two compounds may have distinct mechanisms of action in cancer cells (Table 2). Gemcitabine given before sapacitabine may be more effective than other schedules.

In the doxorubicin/sapacitabine combinations, synergism was observed at high concentrations using sequential exposure, while only additive effects were observed at lower concentrations in both cell lines. Concomitant schedules yielded additive/antagonistic effects (Table 2). Sequential exposure seems to be more effective than combined exposure in both cell lines.

In the oxaliplatin/sapacitabine combinations, various degrees of synergistic activity (CI<1) were observed irrespective of whether sapacitabine was administered before or after oxaliplatin in HCT116. In COLO205 cells, synergy was obtained only when sapacitabine was given concomitantly with oxaliplatin. Therefore, in the colon cancer cell line, COLO205, the effect of the sapacitabine–oxaliplatin combination appeared to be schedule dependent (Table 2).

Combinations of sapacitabine with seliciclib yielded highly synergistic effects for all schedules in the human HCT116 colon cell line, while only additive/antagonistic effects were observed in COLO205 cells. Overall, the optimal schedule appeared to be when seliciclib was given before sapacitabine.

## DISCUSSION

This study aimed at evaluating the effects of sapacitabine in solid tumour cell lines. The activity of sapacitabine was investigated in a panel of cell lines characterised for resistance to anticancer agents, considered as representative of the most commonly observed tumour types. Cytotoxics evaluated in this study were selected based on their mechanisms of action, including nucleoside analogues for evaluation of cross-resistance with ara-C, gemcitabine and CNDAC, as well as based on more pragmatic reasons aiming at comparing cytotoxic effects of sapacitabine with that of already used anticancer agents. Finally, this study aimed at identifying a pharmacological rational for combining sapacitabine with other anticancer agents in further clinical studies including seliciclib, a novel cell cycle inhibitor currently in clinical trials. In our study, it is surely not surprising that sapacitabine and CNDAC may display overlapping cytotoxic effects if the later is a metabolite of the former. However, differences observed between those two compounds may suggest that sapacitabine may act both through its bioconversion into CNDAC and may also act by other mechanisms. Although of major interest, it remains to be demonstrated whether sapacitabine requires to be converted by amidases *in vitro* or can be directly incorporated into cancer cells, undergoing specific metabolic processes. We observed that sapacitabine displayed antiproliferative activity across a broad range of concentrations in a variety of human cancer cell lines, including cancer cells that were shown to be primarily resistant to several anticancer drugs. Moreover, sapacitabine showed cytotoxic activity against dCK-deficient L1210 cells, indicating a dCK-independent mechanism of action. Finally, sapacitabine showed a synergistic effect when combined with gemcitabine and sequence-specific synergy with doxorubicin and oxaliplatin. Based on these observations, sapacitabine appeared as a good candidate for further evaluation in combination with currently used clinical anticancer agents in tumour types with unmet needs.

The antiproliferative effects of sapacitabine in terms of IC_50_ values were better than those observed with CNDAC and ara-C. When comparing the cytotoxic effects of these three pyrimidine analogues, sapacitabine appears to retain activity in several cells that were poorly sensitive to CNDAC and ara-C such as colon HT29 and HCC2998, breast MCF7 and ovarian IGROV1. In contrast, gemcitabine achieved cell growth inhibition at lower concentrations. Our study, as well as previously published data, seems to indicate that both CNDAC and sapacitabine are less potent than gemcitabine in cellular and *in vivo* assays. However this may not turn out to be detrimental in the clinic given that sapacitabine may be given orally and displays additive/synergistic effects with other anticancer agents, allowing maintaining activity in clinical trials. We observed that active concentrations, as assessed by IC_50_ values, ranged from 3.0 to 67.0 *μ*M with colon HCT116 and breast MDA-MB-435 being the most sensitive and resistance cancer cells respectively. Based on animal data and preliminary pharmacokinetic information, this range of concentrations appears to be achievable in future clinical trials in humans. Other authors have demonstrated that sapacitabine also had cytotoxic activity against a broad spectrum of human tumour cells, and although the average cytotoxicities were comparable with CNDAC, sapacitabine was more potent (Hanaoka *et al*, 1999). In gastric, lung, mammary, colon, ovary and epidermoid cell lines, the IC_50_ values for sapacitabine ranged from 0.1 to 11 *μ*M, whereas for CNDAC they ranged from 0.1 to >350 *μ*M (Azuma *et al*, 1993; Hanaoka *et al*, 1999; Matsuda and Sasaki, 2004).

Furthermore, sensitivity profiles of dCK-proficient and -deficient cell lines demonstrated resistance to ara-C and CNDAC when dCK is downexpressed. L1210-dCK**^−^** cells were approximately 2500- and 600-fold more resistant to ara-C and CNDAC than parental L1210-dCK**^+^** cells, respectively. This resistance was not found when cells were incubated with sapacitabine, suggesting a transport-related mechanism of resistance. This finding showing that sapacitabine retained cytotoxic activity against dCK-deficient L1210 cells strongly suggests that sensitivity to sapacitabine may not only be dependent on dCK expression. The role of dCK in sensitivity to CNDAC and sapacitabine has also been observed in MES-SA-10K, a cell line that derives from MES-SA cell deficient in dCK, selected after repeated passages in the presence of gemcitabine (Galmarini and Green, unpublished observations; Jordheim *et al*, 2004). Although dCK-independent mechanisms are not yet finally understood, other enzymes such as CDA have been recently shown to play a role in the mechanism of action of sapacitabine (Fleming *et al*, 2007). These results support the idea that sapacitabine may emerge as a potent and active agent against dCK-deficient cells.

In our experiments, exposure of HCT116 cancer cells to sapacitabine and CNDAC for 48 h led to the accumulation of cells in G_2_ phase of cell cycle. The G_2_ checkpoint is regarded as a resistance mechanism that could permit cells to repair potentially lethal damage such as single-strand breaks. Abrogating this checkpoint reduces the possibility of repair and hence leads to increased cell death. Our data are consistent with the hypothesis that cells treated by sapacitabine or CNDAC may directly progress into G_2_/M phase of cell cycle. Other authors indicated that after incubation with cytostatic concentrations of CNDAC, cell cycle progression was delayed during S phase, but that cells arrested predominantly in the G_2_ phase (Azuma *et al*, 2001; Liu *et al*, 2005). This unique cell cycle arrest pattern was related to the strand-breaking action of CNDAC caused by the *β*-elimination-mediated mechanism. The cytotoxic action of CNDAC may be explained by the fact that human tumour cells are able to accumulate and retain CNDACTP and that the analogue has the ability to terminate further extension.

To further understand the basis of sapacitabine and CNDAC activity in these cell lines of differing origin, we measured mRNA levels of several enzymes commonly implicated in resistance to nucleoside analogues such as dCK, cN-II, POL and hENT1. Colorectal, breast and the HOP92 lung cancer cell lines commonly demonstrated high expression levels of hENT1, low-medium levels of dCK and variable expression of both cN-II and DNA polymerase, and HOP62 lung and ovarian cancer cell lines showed low expression of the transporter hENT1 and medium cN-II and DNA polymerase expression; however, no obvious correlation between drug sensitivity and mRNA expression levels of these metabolic factors was identified. Our results are in contrast to those described by Matsuda and Sasaki (2004). These authors demonstrated that levels of expression of metabolic enzymes such as dCK or cytidine deaminase influenced the antiproliferative effects of CNDAC. Moreover, expression and/or function of these factors appear to be influenced by the activation of various oncogenes (Zhang *et al*, 1999).

To further improve the effects of sapacitabine, circumvent potential resistance to this agent, and find combinations that may be used in clinical trials, we investigated a number of sapacitabine combinations using both simultaneous and sequential exposures to drugs. Combination studies demonstrated synergistic effects when sapacitabine was combined with compounds such as oxaliplatin, gemcitabine, docetaxel and doxorubicin over a broad range of concentrations in human colon cancer cells using several administration schedules. Interestingly, synergy might be sequence-dependent for oxaliplatin and docetaxel. Synergism between sapacitabine and gemcitabine strongly suggests that although closely related these two compounds have distinct mechanisms of action in cancer cells. Phase I clinical data have shown that after oral administration, sapacitabine was not detectable in urine suggesting that most of sapacitabine is converted into CNDAC in human (Delaunoit *et al*, 2006). In our experiments, sapacitabine was consistently more cytotoxic than CNDAC leading to combination studies using sapacitabine instead of CNDAC. Although combination with CNDAC was not performed, it is unlikely that results may have been superior with CNDAC than that of sapacitabine in our models.

In summary, our study provides additional data supporting further preclinical and clinical pharmacological investigations of sapacitabine in human cancers. Our results suggest that sapacitabine possesses an interesting toxicity profile compared to several other anticancer agents including other pyrimidine analogues. Moreover, sapacitabine showed cytotoxic activity against dCK-deficient cell lines indicating that this compound can be active in cells resistant to CNDAC, ara-C and other nucleoside analogues. Our preclinical data showed that sapacitabine combinations yield synergistic activity with other anticancer agent (including gemcitabine) supporting further evaluation of those combinations both simultaneously and sequentially in clinical trials.

## Figures and Tables

**Figure 1:**
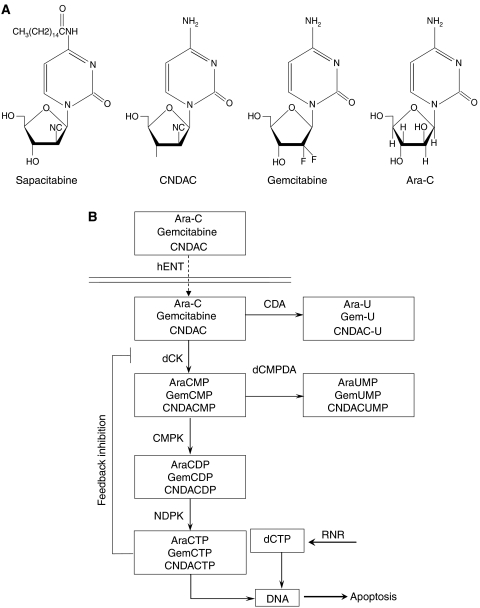
(**A**) Chemical structure of sapacitabine, CNDAC, gemcitabine and ara-C; (**B**) Metabolism of ara-C, gemcitabine and CNDAC. Nucleosides enter the cell via the human equilibrative nucleoside transporter 1 (hENT1). Inside the cell, they are phosphorylated to monophosphates by deoxycytidine kinase (dCK). Ara-CMP and CNDACMP are subsequently phosphorylated to ara-CTP and CNDACTP, the active metabolites. Incorporation of CTPs into DNA blocks DNA synthesis and leads to cell death. Ara-CTP formation can be obstructed. Cytidine deaminase (CDA) and deoxycytidylate deaminase (dCMPD) convert ara-C to ara-Uridine (U), and ara-CMP to ara-UMP, respectively. dCTP can be synthesised directly via the *de novo* pathway by ribonucleotide reductase (RNR).

**Table 1:** Antiproliferative effects (IC_50_ using MTT assay) of sapacitabine, CNDAC, seliciclib and several anticancer compounds in a panel of human and murine cancer cell lines

		**IC_50_ (*μ*M)**									
**Tumour type**	**Cell line**	**Sapacitabine**	**CNDAC**	**Gemcitabine**	**Ara-C**	**Seliciclib**	**Doxorubicin**	**Docetaxel**	**Cisplatin**	**Oxaliplatin**	**5-FU**
Colon	HT29	4.5±0.7	160±43	0.015±0.003	130±40	19±3	1.07±0.16	0.01±0.002	25±3	60±12	24±1
	HCT116	3.0±0.6	2.8±0.6	0.05±0.01	1.2±0.4	11±2	0.10±0.02	0.01±0.02	9.2±0.9	20±4	5.3±1.3
	HCC2998	3.5±0.8	110±21	0.01±0.002	3.7±0.5	23±5	0.70±0.14	0.004±0.001	25±5	4±1.4	10±1
	COLO205	5.0±0.8	8±2	0.2±0.1	28±8	12±3	1.5±0.5	0.006±0.001	9.5±2.5	8±1.6	240±20
											
Breast	MCF7	7.0±1.8	850±60	0.01±0.002	>300	16±3	0.7±0.1	0.01±0.004	32±2	35±7	10±1
	MDA-MB-435	67±14	95±13	0.04±0.008	38±9	19±4	2.0±0.4	0.01±0.002	13±2.6	37±21	10±2
											
Lung	HOP62	6.0±1.7	7.0±1.8	0.01±0.002	6.3±2.8	8±2	0.14±0.04	0.02±0.04	9.3±0.9	200±40	118±32
	HOP92	38±8	23±6	0.02±0.004	1.3±0.3	26±4	0.10±0.05	0.005±0.001	4.4±0.4	1.4±0.2	11±1
											
Ovarian	IGROV1	6.5±1.6	89±12	0.02±0.004	89±21	38±4	1.3±0.3	0.02±0.01	26±6	107±21	29±1
	OVCAR1	45±7	>900	1.2±0.2	>300	20±6	1.0±0.2	50±0.12	12.10±3.93	50±10	24±1
											
Leukaemia	L1210	20±6	1.7±0.3	ND	0.08±0.02	ND	ND	ND	ND	ND	ND
	L1210dCK-	29±4	1000±150	ND	>100	ND	ND	ND	ND	ND	ND

ND=not determined.

**Table 2:** Summary of effects of combination agent study using five anticancer agents in combination with sapacitabine in three treatment schedules

		**CI, mean (SD)**							
		**COLO205**				**HCT116**			
**Combination schedule**		**ED <0.25**	**ED 0.25–0.50**	**ED 0.50–0.75**	**ED >0.75**	**ED <0.25**	**ED 0.25–0.50**	**ED 0.50–0.75**	**ED >0.75**
Docetaxel-based	48 h Sapa → 24 h Doc	—	—	1.8 (1.2)	1.0 (0.6)	7.4 (5.0)	0.9 (0.1)	0.9 (0.4)	1.3 (0.5)
combinations	24 h Doc → 48 h Sapa	—	0.5 (0.1)	1.2 (0.1)	0.5 (0.3)	2.1 (1.1)	0.7 (0.1)	0.8 (0.1)	0.1 (0.1)
	48 h Sapa+Doc	1.3 (0.1)	1.5 (0.7)	0.9 (0.2)	0.5 (0.1)	—	1.5 (0.1)	0.7 (0.3)	0.7 (0.4)
									
Gemcitabine-based	48 h Sapa → 24 h Gem	1.4 (0.5)	1.0 (0.1)	1.0 (0.4)	1.2 (0.8)	2.6 (0.9)	—	0.7 (0.1)	1.3 (0.8)
combinations	24 h Gem → 48 h Sapa	1.5 (0.3)	1.0 (0.3)	0.5 (0.1)	0.4 (0.2)	—	0.4 (0.1)	0.5 (0.3)	0.4 (0.3)
	48 h Sapa+Gem	6.0 (2.0)	1.7 (0.8)	1.0 (0.3)	0.6 (0.5)	—	—	1.3 (0.9)	0.4 (0.2)
									
Doxorubicin-based	48 h Sapa → 24 h Dox	—	—	—	0.6 (0.5)	—	1.1 (0.2)	0.8 (0.4)	0.7 (0.5)
combinations	24 h Dox → 48 h Sapa	—	—	—	0.7 (1.1)	0.8 (0.1)	0.8 (0.3)	1.1 (0.5)	0.6 (0.6)
	48 h Sapa+Dox	—	2.0 (1.9)	1.2 (0.9)	0.7 (0.7)	1.9 (0.9)	1.0 (0.3)	1.2 (0.2)	1.0 (0.3)
									
Oxaliplatin-based	48 h Sapa → 24 h Oxa	—	—	—	1.5 (0.8)	8.0 (1.2)	0.4 (0.1)	0.3 (0.1)	0.6 (0.4)
combinations	24 h Oxa → 48 h Sapa	—	1.7 (0.9)	1.4 (1.2)	0.6 (0.4)	1.8 (0.6)	0.5 (0.4)	0.5 (0.4)	0.4 (0.4)
	48 h Sapa+Oxa	—	1.2 (0.7)	1.0 (0.3)	0.2 (0.1)	2.4 (1.0)	1.1 (0.2)	0.7 (0.2)	0.7 (0.7)
									
Seliciclib-based	48 h Sapa → 24 h Sel	—	2.2 (1.1)	1.8 (0.5)	1.3 (0.5)	—	—	1.4 (1.2)	0.5 (0.3)
combinations	24 h Sel → 48 h Sapa	1.2 (0.3)	1.4 (0.1)	0.9 (0.2)	0.6 (0.1)	—	0.8 (0.2)	0.7 (0.1)	0.7 (0.5)
	48 h Sapa+Sel	—	—	0.7 (0.6)	0.8 (0.4)	—	1.0 (0.1)	0.8 (0.3)	0.7 (0.3)

CI=combination indices; Doc=docetaxel; Dox=doxorubicin; ED=fraction of cells affected by drug combination; Gem=gemcitabine; Oxa=oxaliplatin; Sapa=sapacitabine; Sel=seliciclib.
